# Initiation of antiretroviral therapy or antiretroviral prophylaxis in pregnant women living with HIV registered in five townships of Mandalay, Myanmar: A cross sectional study

**DOI:** 10.1186/s12884-019-2627-6

**Published:** 2019-12-05

**Authors:** Khine Wut Yee Kyaw, Aye Aye Mon, Khaing Hnin Phyo, Nang Thu Thu Kyaw, Ajay M. V. Kumar, Than Than Lwin, Zaw Zaw Aung, Thet Ko Aung, Myo Minn Oo, Thurain Htun, Sai Soe Thu Ya, Srinath Satyanarayana, Htun Nyunt Oo

**Affiliations:** 1 Department of Operational Research, International Union Against Tuberculosis and Lung Disease (The Union), Mandalay, Myanmar; 2 HIV unit, International Union Against Tuberculosis and Lung Disease (The Union), Mandalay, Myanmar; 3 Center for Operational Research, International Union Against Tuberculosis and Lung Disease (The Union), Paris, France; 4 International Union Against Tuberculosis and Lung Disease (The Union), South-East Asia Office, New Delhi, India; 5 Yenepoya Medical College, Yenepoya (Deemed to be University), Mangaluru, India; 6 National AIDS Programme, Department of Public Health, Ministry of Health and Sports, Nay Pyi Taw, Myanmar; 7 Monitoring, Evaluation, Accountability and Learning Unit (HIV), International Union Against Tuberculosis and Lung Disease (The Union), Mandalay, Myanmar

**Keywords:** Myanmar, PMTCT, operational research, linkage, HIV

## Abstract

**Background:**

A series of interventions are required to prevent mother to child transmission (PMTCT) of Human Immunodeficiency Virus (HIV) starting from HIV testing of pregnant women, initiating antiretroviral therapy (ART) or antiretroviral prophylaxis to HIV-positive pregnant women to providing HIV prophylaxis to newborn babies. Gaps in each step can significantly affect the effectiveness of PMTCT interventions. We aimed to determine the gap in initiation of ART/antiretroviral prophylaxis for pregnant women living with HIV, delay in initiation of ART/antiretroviral prophylaxis and factors associated with the delay.

**Methods:**

This is a cross sectional study using routinely collected programme data from five health facilities providing PMTCT services located at Township Health Departments (THD) of Mandalay, Myanmar.

**Results:**

There were 363 pregnant women living with HIV enrolled between January 2012 and December 2017. Sixty (16%) women were excluded from the study due to missing data on dates of HIV diagnosis. Of 303 (84%) women included in the study, 89/303 (29%) and 214/303 (71%) were diagnosed with HIV before and during current pregnancy respectively. Among 214 women, 180 (84%) women were started on ART by the censor date (31st March 2018). Among those who started ART, 109 (61%) women had a delay of starting ART > 2 weeks from diagnosis. Women residing in township 4 had a significantly higher risk of delay in initiation of ART/antiretroviral prophylaxis compared to women residing in township 1 [adjusted prevalence ratio 4.2 (95% confidence interval 1.2–14.8].

**Conclusions:**

We found that one in four women living with HIV knew their HIV status before current pregnancy. Although the rate of ART/antiretroviral prophylaxis initiation was high among pregnant women living with HIV, there was a delay. Early initiation of ART/antiretroviral prophylaxis among newly HIV diagnosed pregnant women needs to be strengthened.

## Background

Prevention of mother to child transmission (PMTCT) of Human Immunodeficiency Virus (HIV) services include providing maternal antiretroviral therapy (ART)/ antiretroviral prophylaxis and infant’s antiretroviral prophylaxis. This service can dramatically reduce the transmission of HIV infection to less than 2% in non-breastfed infants and less than 5% in breastfed infants and these levels of reductions are also the targets for elimination of mother to child transmission (eMTCT) [1–6].

Prevention of HIV transmission from pregnant women living with HIV to their infants requires sequential interventions starting from HIV testing of pregnant women to providing antiretroviral prophylaxis to newborn babies. Gaps in each step of interventions can significantly influence the effectiveness of PMTCT interventions. A study in Kenya showed that only 17 and 35.4% of pregnant women tested HIV-positive referred from each of the two government hospital antenatal care services were enrolled in HIV care within 6 months [7]. World Health Organization (WHO) recommends early ART initiation in HIV-positive women (as soon as possible) as an effective intervention in reducing mother to child transmission of HIV and studies show that at least 4–13 weeks of ART is required to achieve viral suppression at the time of delivery [2, 8]. However, a study conducted in Cape Town between 2003 and 2010 showed that there was a delay in initiation of ART (21 days after HIV diagnosis) among pregnant women attending antenatal care services [9]. WHO published updated guideline in 2016 which recommended provision of lifelong ART to HIV-positive pregnant women regardless of WHO staging and CD4 count which can minimize or remove the delay in initiation of ART without waiting for the CD4 results and deterioration of patients’ clinical condition [10].

In Myanmar, HIV prevalence among adult population aged ≥15 years was estimated at 0.59% in 2015 [11]. Since 2005, antiretroviral drugs for PMTCT were available and provided to pregnant women [11]. At the end of 2015, PMTCT services including HIV counselling and testing and referral or onsite ART was established by National AIDS Programme (NAP) in 304 townships and 38 hospitals in collaboration with Maternal and Reproductive Health Division (MRH) and Child Health Division (CHD) [11].

According to UNAIDS estimates, there were 5100 pregnant women living with HIV in 2015 in Myanmar [12]. About 7% of these pregnant women (~ 370) women lived in Mandalay region [13]. An assessment report in Myanmar mentioned that only 59% of women living with HIV accessed antiretroviral prophylaxis between 2012 and 2014 [13]. Further, the rate of initiation of ART/antiretroviral prophylaxis among women enrolled to facilities providing PMTCT services in township health department (THD) have not yet been documented in this setting. In this study, we aimed to estimate the delay in initiation of ART/antiretroviral prophylaxis and the factors associated with delay among pregnant women living with HIV and enrolled in township health facilities providing PMTCT services.

## Methods

### Study design

This was a cross sectional study involving secondary analysis of patient records.

### Setting

The study was conducted in five health facilities providing PMTCT services and are jointly operated by the National AIDS Programme (NAP), the township medical team and the International Union Against Tuberculosis and Lung Disease (The Union). The Union is an international non-governmental organization and has been implementing integrated HIV care programme (IHC) since 2005 in collaboration with NAP, Department of Public Health, Ministry of Health and Sports, in Myanmar. The IHC clinic has been providing PMTCT services in Central Women Hospital (CWH) and Mandalay Teaching Hospital (MTH) in Mandalay since March 2011. Pregnant women from THD are referred to CWH or MTH for HIV care including ART/antiretroviral prophylaxis.

**Fig. 1:**
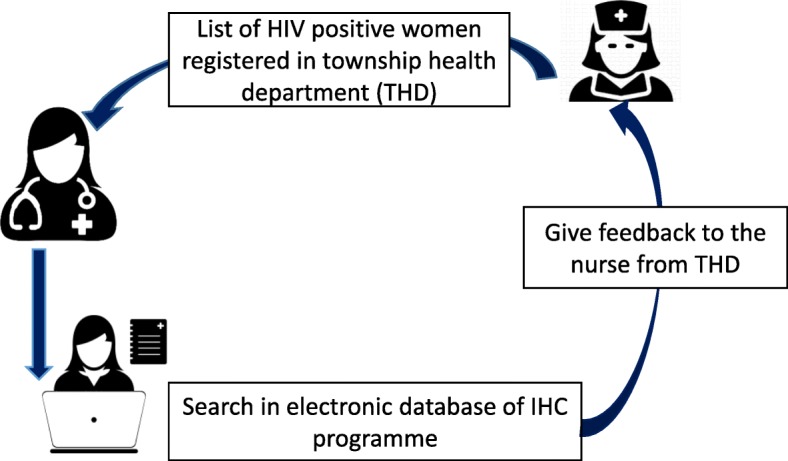
Flow of updating the women’s ART status between PMCT focal person* and medical doctor¥ 17: ART = antiretroviral therapy, PMTCT = prevention of mother to child transmission, *PMTCT focal person from the township health department, ¥Medical doctor from The Union. (The Doctor icon and Medical record icon were created by Wilson Joseph from the Noun Project [www.thenounproject.com] and the Nurse icon was created by Pixel perfect from www.flaticon.com)

There is a responsible medical doctor assigned by The Union to support PMTCT focal person at THD for documenting linkage of HIV positive pregnant mother to HIV care. The medical doctor visits THD every week, gets the list of HIV positive women newly registered in township PMTCT register and searches those patients in electronic database of IHC clinic at CWH or MTH. Then the information on enrolment to HIV care and ART/antiretroviral prophylaxis initiation status of pregnant women is shared back to the PMTCT focal person of THD and the linkage information such as IHC code and ART/antiretroviral prophylaxis initiation date are recorded in PMTCT register. The routine procedure of updating the women’s ART status is shown in Fig. 1.

**Fig. 2:**
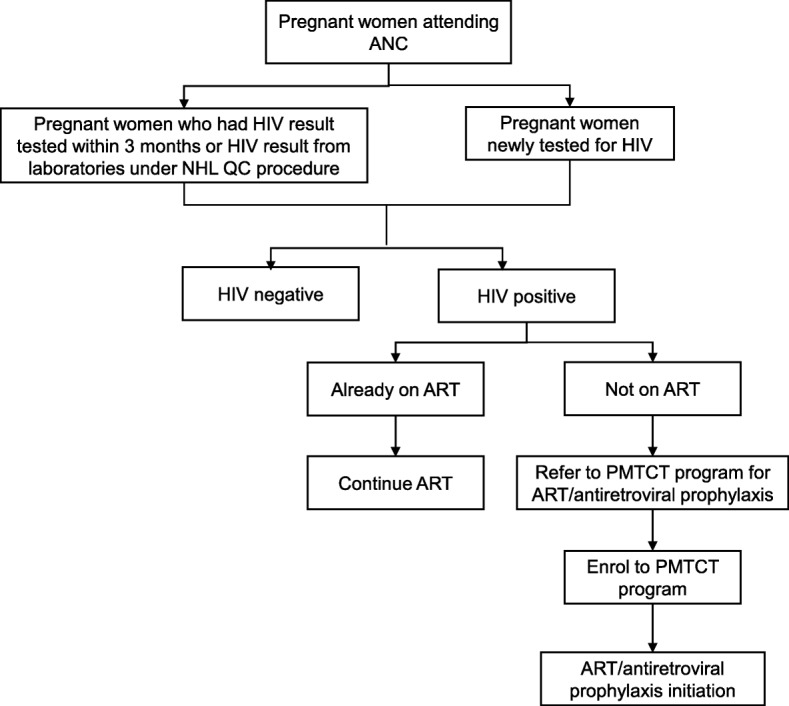
Flow of pregnant women attending ANC at the township health department in Mandalay: ANC; Antenatal care, HIV; Human immunodeficiency virus, NHL; National Health Laboratory, QC; quality control, ART; antiretroviral therapy, PMTCT; prevention of mother to child transmission

The PMTCT focal person of THD does the following activities. During first ANC visit in THD, pregnant women are given ANC code and baseline demographic characteristics along with previous medical history are recorded in the ANC registers. The women undergo HIV counselling and testing if they have not undergone this test within the past 3 months or if they have undergone this test from non-National Health Laboratory (NHL) accredited laboratories. The pregnant women who test positive were referred to CWH or MTH for enrolment to HIV care, CD4 testing and provision of ART/antiretroviral prophylaxis according to national guidelines prevailing during the study period [2, 14–16]. A diagram illustrating the flow of patients is shown in Fig. 2.

The ART was provided if the CD4 cell count was < 350 cells/mm^3^ before 2015, < 500 cells/mm^3^ from 2015 to 2016 and it was changed to ‘all’ regardless of CD4 count after 2016. In different time periods, different antiretroviral prophylaxis was provided to women with CD4 count higher than cutoff points: 1) Before 2013: PMTCT option A wherein eligible pregnant women received Zidovudine (AZT) only; 2) Between 2011 and 2014: PMTCT option B wherein eligible pregnant women received triple drug ART throughout pregnancy, at the time of delivery and stopped ART a week after discontinuation of breast feeding, 3) Between 2014 and 2016: PMTCT option B+ wherein eligible pregnant women received triple drug ART during pregnancy, delivery and then continued the ART for life. Between 2011 and 2013, the attending physicians based on their own discretion either proved option A or option B to the eligible pregnant women.

The description of care and services provided in IHC clinic at CWH has been reported elsewhere [17]. In brief, comprehensive PMTCT services include care and treatment provided by specialists from the hospital, medical officers employed by The Union, PLHIV network and medical social workers from the hospital. Pregnant women are scheduled for follow up visit periodically. After delivery, mother and infant(s) are followed up until 18th month post-partum. If the exposed infant is diagnosed as HIV positive, then the infant is transferred out to pediatric IHC clinic for ART initiation and the mother to adult clinic for further follow-up. If the infant is declared to have no infection, the mother is then transferred to the adult ART clinic, and the infant is discharged from further follow-up.

In THD ANC clinics, the responsible PMTCT focal person records patient information in standardized PMTCT register. At IHC clinic, the visit forms are filled by trained medical doctors and these forms are transcribed into an electronic database of NAP and The Union’s IHC programme after each clinic by trained data entry staff.

### Study population

Pregnant women living with HIV enrolled in five study sites at THDs were included. From the first two study sites, pregnant women who were enrolled between January 2012 and December 2017 were included. From the other two study sites, those who were enrolled between January 2013 to December 2017 and from the last study site, those who enrolled between January 2015 and December 2017 were included in the study. The study cohort was followed up until 31st March 2018 (censor date).

### Source of data, data variables

Data from the paper-based PMTCT registers was single entered into EpiData database (version 3.1) by trained data staff in April 2018. Information related to HIV care at IHC clinic (CWH and MTH) was available in the NAP-Union database. The study variables included: name, registered township, age, HIV diagnosis date, ART/antiretroviral prophylaxis initiation date, date of delivery, baseline CD4 count, baseline WHO staging, last menstrual period (LMP), employment status.

The two electronic databases were linked using IHC code. The women who did not have IHC programme code in PMTCT register were manually searched in IHC electronic database using name and age. The distances in google map were used to calculate the distance between enrolled PMTCT clinics and registered townships for women enrolled in HIV care. The last menstrual date was calculated from date of delivery by subtracting 280 days from date of delivery for those whose LMP was not recorded in electronic database. Delayed initiation of ART/antiretroviral prophylaxis was defined as being initiated on ART/antiretroviral prophylaxis after 2 weeks from the date of HIV diagnosis.

### Statistical Analysis

Electronic databases were imported into STATA version 14.2 (Stata Corp. College Station, TX, USA). Data were anonymized and de-identified prior to analysis. Median (interquartile range-IQR) was used for summarizing continuous variables and numbers and proportions were used for summarizing categorical variables. Time to ART initiation was calculated by subtracting the date of HIV diagnosis and date of treatment initiation among women diagnosed with HIV during current pregnancy and initiated on ART/antiretroviral prophylaxis.

To depict the distribution of time to event, Kaplan-Meier curves were plotted. The unadjusted and adjusted prevalence ratio (PR) were calculated for the delay in initiation of ART/antiretroviral prophylaxis by multivariable binomial log regression models (multivariable Poisson regression models with robust standard error estimates if the binomial models failed to achieve convergence). A *P*-value of less than 0.05 was considered statistically significant for all analyses.

## Results

**Fig. 3:**
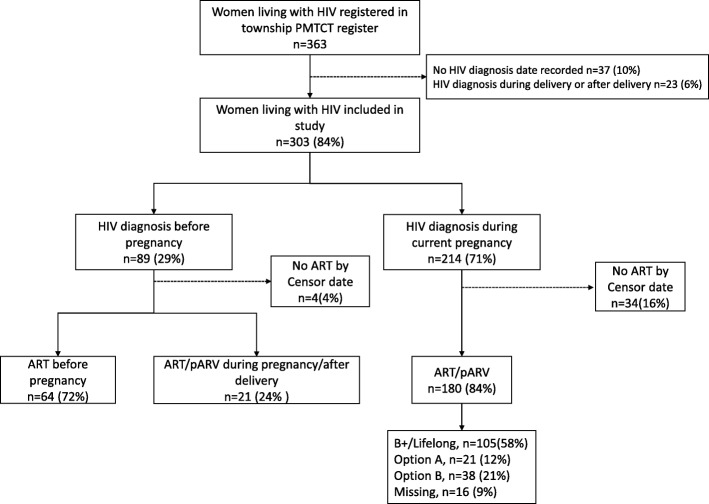
Cascade of care among pregnant women living with HIV registered* between 2012 and 2017: HIV; Human immunodeficiency virus, PMTCT; prevention of mother to child transmission; censor date; 31st March 2018, ART; antiretroviral therapy, antiretroviral prophylaxis; antiretroviral prophylaxis, *registered in five township health department in Mandalay

There were 363 pregnant women living with HIV registered in the study sites, of whom, 303 (84%) women were included in the analysis. Sixty women (16%) were excluded because 37/363 (10%) of them did not have HIV diagnosis date and 23/363 (6%) were diagnosed with HIV during or after delivery. The median (IQR) age was 29 (25–32) years. Of 303 women, 89 (29%) were diagnosed with HIV before current pregnancy and 214 (71%) were diagnosed during pregnancy. Figure 3 shows the cascade of care with losses at each step of PMTCT services. The CD4 count at enrolment to HIV care was available for 296 women and median (IQR) baseline CD4 count was 337 (238–475) cells/mm^3^.

### Initiation of ART/antiretroviral prophylaxis

Among women diagnosed with HIV during their current pregnancy, 180/214 (84%) were initiated on ART/antiretroviral prophylaxis. The median (IQR) duration between HIV diagnosis and initiation of ART/antiretroviral prophylaxis was 20 (10–51) days. Only 6/180 (3%) of them initiated ART/antiretroviral prophylaxis on the same day as HIV diagnosis. The 105/180 (58%) women were initiated on lifelong/option B+, 21/180 (12%) were on option A, 38/180 (21%) were on option B and 16/180 (9%) had no protocol type recorded.

### Delay in initiation of ART/antiretroviral prophylaxis

**Fig. 4:**
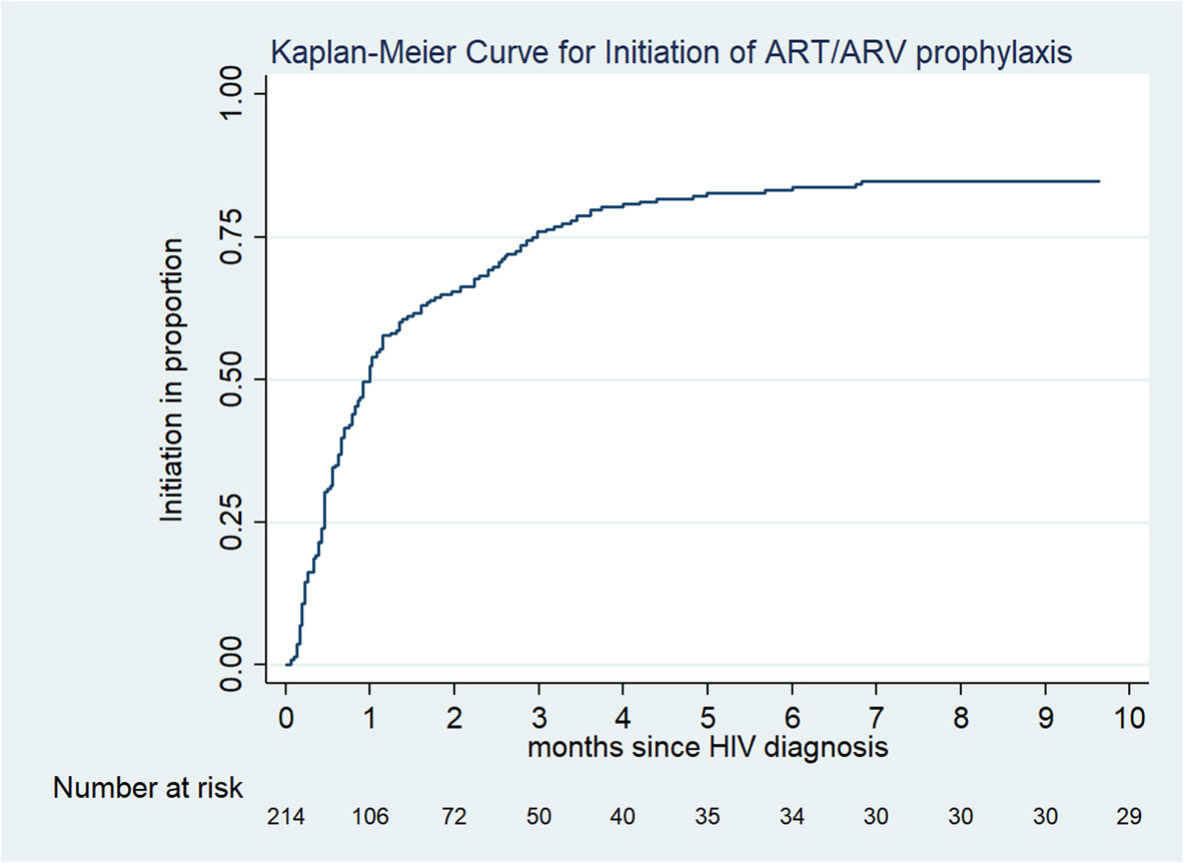
Kaplan-Meier-Curve for time to ART/antiretroviral prophylaxis Initiation among women diagnosed with HIV during current pregnancy: ART = antiretroviral therapy, HIV=Human Immunodeficiency Virus

The time to initiating ART/antiretroviral prophylaxis among women diagnosed with HIV during current pregnancy is described in Fig. 4. Of 180 women initiated on ART/antiretroviral prophylaxis, 109 (61%) were initiated after 2 weeks from HIV diagnosis date (defined as delay in this study). Women resident in township 4 had a significantly higher risk of delay in initiation of ART/antiretroviral prophylaxis compared to township 1 (names of the townships have not been disclosed in the paper to maintain confidentiality) with the adjusted prevalence ratio, aPR [95% confidence interval, CI] of 4.2 [1.2–14.8]. (Table 1).

**Table 1:** Factors associated with delayed ART/antiretroviral prophylaxis initiation among women diagnosed with HIV during their current pregnancy

Description	total	col %	delay^¥^	row %	PR (95% CI)	aPR(95% CI)
Total	180		109	61%		
age group						
< =20	9	5%	5	56%	1	
21–30	112	62%	66	59%	1.1 (0.6–1.9)	
> =31	59	33%	38	64%	1.2 (0.6–2.1)	
township						
Township 1	11	6%	2	18%	1	1
Township 2	63	35%	37	59%	3.2 (0.9–11.5)	3.6 (0.8–15.5)
Township 3	16	9%	6	38%	2.1 (0.5–8.4)	2.4 (0.5–11.8)
Township 4	58	32%	43	74%	4.1 (1.1–14.5)*	4.2 (1.2–14.8)*
Township 5	32	18%	21	66%	3.6 (1.0–13.0)	3.6 (1.0–13.0)
CD4 count						
< 350	100	56%	64	64%	1.1 (0.9–1.5)	
≥ 350	73	41%	41	56%	1	
Missing	7	4%	4	57%		
WHO staging						
Stage I/II	154	86%	91	59%	1	
Stage III/IV	15	8%	11	73%	1.2 (0.9–1.7)	
Missing	11	6%	7	64%		
Occupation						
Yes	63	35%	38	60%	1	
No	87	48%	53	61%	1 (0.8–1.3)	
Missing	30	17%	18	60%		
Distance						
≤6 km	69	38%	36	52%	1	1
> 6 km	100	56%	66	66%	1.3 (1–1.7)	1.2 (0.5–2.5)
Missing	11	6%	7	64%		

*ART/antiretroviral prophylaxis* Antiretroviral therapy/Antiretroviral prophylaxis, *col%* column percentage, *PR* prevalence ratio, *95% CI* 95% confidence interval, *aPR* adjusted prevalence ratio, ^¥^ delay initiation of ART/antiretroviral prophylaxis, *km* kilometer, *p value< 0.05, *p*-value < 0.2 were included in adjusted analysis, Delayed intiation of ART/antiretroviral prophylaxis = more than 2 week from date of HIV dagnosis.

## Discussion

There are three important findings in this study. First, one fourth of the women were diagnosed with HIV before current pregnancy. Second, the ART uptake was very high. Third, there was a delay in initiation of ART/antiretroviral prophylaxis and the delay was longer in township 4.

One-fourth of the pregnant women were tested HIV positive before their current pregnancy. We do not have information on whether these pregnancies were planned/desired or unintended. If those are unintended pregnancies, the HIV care programme should strengthen the family planning among PLHIV by encouraging the consistent use of appropriate contraceptives as recommended in National guideline [16]. On the other hand, if the pregnancy was intended/planned, there are ways to assist women living with HIV to fulfil their desire of giving birth to a child while minimizing the risk of mother to child transmission (MTCT) [18, 19]. In Africa, 30 to 80% of women living with HIV have expressed their desire to give birth to a child [20–23]. Hence, it is important to have a standard operational procedure in the HIV clinic for PLHIV planning to have children.

There are various strategies for safe conception such as ART, pre-exposure prophylaxis, timed unprotected intercourse, manual/self-insemination, sperm washing, and voluntary male medical circumcision (VMMC) [18]. In Myanmar, NAP recommends the initiation of ART in all PLHIV regardless of WHO staging and at any CD4 count which can be one of the strategies to reduce MTCT of HIV [14]. All medical doctors should be sensitized to follow this recommendation. PLHIV should also be made aware of this policy so as to increase the demand for services.

The ART uptake among pregnant women was very high in our study. The study in Cape Town shows 55, 38 and 45% of eligible women were initiated on ART at three different sites [24]. There are common barriers to initiate ART reported in previous studies conducted in Tanzania and Kenya such as not understanding the importance of HIV care, being too ill to attend, disclosure related issues, wanting to wait until delivery, long distances to the HIV clinic, stigma, denial, poor health services and lack of money [25, 26]. All the pregnant women in our setting were eligible for initiating either ART or antiretroviral prophylaxis with different eligibility criteria. There was a small proportion of pregnant women who did not start ART/antiretroviral prophylaxis by the end of the study period. Barriers for initiating ART/antiretroviral prophylaxis among them needs further exploration.

A systematic review on the impact of different interventions on enrolment to HIV care reported that integration to ANC services, intensified post-test counselling and home visit by peer supporter led to better linkage to care [27]. In our setting, tracking of women referred to HIV care was done by a medical officer employed by The Union and if the HIV positive women had not yet enrolled to care, the medical officer would inform the township PMTCT focal person to trace the woman via basic health staff and encourage enrolling into care. This system could have played an important role in minimizing dropout.

In this study, women living in township 4 experienced longer delays in initiation of ART/antiretroviral prophylaxis. We will report this finding to the responsible person of that township. Although the barriers to initiate ART early were not explored in this study, some qualitative studies reported that the pregnant women were afraid of ART side effects, did not have opportunities to ask questions about drugs and dosage during counselling, had heard of someone’s negative experience on ART and received poor partner or communities support [28, 29]. In addition, health system-related factors such as poor provider– patients interaction, staff shortages and service accessibility were reported as barriers for early ART initiation in pregnant women in other settings [30]. Studies also reported that desire to have HIV-free baby and getting support from partner and family members were enablers for a mother to initiate ART at the recommended time [28, 30]. Hence, specific barriers in referral and linkage to HIV care for initiation of ART/antiretroviral prophylaxis in the study setting should be explored and addressed accordingly.

### Strengths

We used routinely collected programme data and the findings reflect ground-level realities. In addition, there is a system of routine data quality assurance in our programme and the data errors are regularly checked and corrected. The data recorded at township department were routinely updated by nurse and The Union staff assisted in updating the information on linkage to HIV care and ART/antiretroviral prophylaxis status. Therefore, the data errors are likely to be minimal and the gap in linkage detected in this study highly likely to reflect reality.

### Limitations

Some important variables for PMTCT service such as gestational age at first ANC visit and date of first ANC visit were not recorded in township PMTCT registers and hence these variables could not be included in this study as this was a retrospective study. We observed a significant amount of missing data in LMP in the IHC excel database. Hence, we derived LMP from the date of delivery and included this in the analysis for estimating the timing of HIV diagnosis with respect to the current pregnancy.

### Areas for future research

First, the magnitude of unintended pregnancies among women who were already HIV positive was not explored in this study. Second, the reasons for delays in initiation of ART/antiretroviral prophylaxis were not explored in this study. Third, the proportion of pregnant women tested for HIV was unavailable. These areas require further evaluation using both quantitative and qualitative research methods.

## Conclusions

We found that one in four women living with HIV knew their HIV status before their current pregnancy. There was high uptake of ART among HIV-positive pregnant women. But, there was delayed initiation of ART/antiretroviral prophylaxis. We recommend that additional variables such as gestational age at first ANC, date of first ANC visit and LMP should be included in the recording system to monitor the PMTCT services more effectively. Early initiation of ART/antiretroviral prophylaxis among pregnant women living with HIV enrolled in township PMTCT care should be strengthened.

## Data Availability

The data contains sensitive HIV patients’ information that was obtained from the Myanmar’s National AIDS Programme after approval from the relevant authorities and in country ethics committee. We have permission to share only aggregate (or pooled) analysed data but not individual patient wise data. Therefore, the data cannot be made available publicly. However, if anyone is interested in accessing the individual patient wise de-identified data, they are requested to contact the corresponding author. The corresponding author will direct them to obtain permission from National AIDS Programme prior to sharing the de-identified individual patient data.

## Abbreviations

AIDS: Acquired Immune Deficiency Syndrome
ANC: Antenatal care
antiretroviral prophylaxis: Antiretroviral prophylaxis
aPR: Adjusted Prevalence ratio
ART: Antiretroviral therapy
col %: Column percentage
CWH: Central Women Hospital
eMTCT: Elimination of mother to child transmission of HIV
HIV: Human Immunodeficiency Virus
IHC: NAP and The Union’s Integrated HIV care programme
Km: Kilometre
LMP: Last Menstrual Period
MTCT: Mother to child transmission
MTH: Mandalay Teaching Hospital
NAP: National AIDS programme
NHL: National Health Laboratory
PLHIV: People living with HIV
PMTCT: Prevention of mother to child transmission
PR: Prevalence ratio
PrEP: Pre-exposure prophylaxis
THD: Township Health Department
